# Leaf Vein Morphological Variation in Four Endangered Neotropical *Magnolia* Species along an Elevation Gradient in the Mexican Tropical Montane Cloud Forests

**DOI:** 10.3390/plants10122595

**Published:** 2021-11-26

**Authors:** Ernesto C. Rodríguez-Ramírez, Leccinum J. García-Morales, Othón Alcántara-Ayala, J. Antonio Vázquez-García, Isolda Luna-Vega

**Affiliations:** 1 Laboratorio de Dendrocronología, Universidad Continental, Urbanización San Antonio, Avenida San Carlos 1980, Junín, Huancayo 12000, Peru; 2 Laboratorio de Biogeografía y Sistemática, Facultad de Ciencias, Universidad Nacional Autónoma de México, Mexico City 04510, Mexico; lexgarcia@yahoo.com (L.J.G.-M.); othon@ciencias.unam.mx (O.A.-A.); 3 Laboratorio de Ecosistemática, Instituto de Botánica, Departamento de Botánica y Zoología, Centro Universitario de Ciencias Biológicas y Agropecuarias, Universidad de Guadalajara, km 15.5 Carr. Guadalajara-Nogales, Camino Ing. Ramón Padilla Sánchez 2100, Nextipac, Zapopan 45221, Mexico; talaumaofeliae@gmail.com

**Keywords:** conservation, climate change, endangered tree species, hydric deficit, leaf vein

## Abstract

Climatic variations influence the adaptive capacity of trees within tropical montane cloud forests species. Phenology studies have dominated current studies on tree species. Leaf vein morphology has been related to specific climatic oscillations and varies within species along altitudinal gradients. We tested that certain Neotropical broad leaf *Magnolia* species might be more vulnerable to leaf vein adaptation to moisture than others, as they would be more resilient to the hydric deficit. We assessed that leaf vein trait variations (vein density, primary vein size, vein length, and leaf base angle) among four *Magnolia* species (*Magnolia nuevoleonensis*, *M. alejandrae*, *M. rzedowskiana*, and *Magnolia vovidesii*) through the Mexican Tropical montane cloud forest with different elevation gradient and specific climatic factors. The temperature, precipitation, and potential evaporation differed significantly among *Magnolia* species. We detected that *M*. *rzedowskiana* and *M*. *vovidesii* with longer leaves at higher altitude sites are adapted to higher humidity conditions, and that *M*. *nuevoleonensis* and *M*. *alejandrae* inhabiting lower altitude sites are better adjusted to the hydric deficit. Our results advance efforts to identify the *Magnolia* species most vulnerable to climate change effects, which must focus priorities for conservation of this ecosystem, particularly in the Mexican tropical montane cloud forests.

## 1. Introduction

Climate variations trigger morphological leaf variations in the Tropical montane cloud forest (TMCF) [1,2]. A range of morphological evidence strongly suggests that leaf vein morphology is adapted to climate [3]. Intraspecific foliar vein variation in high moisture environments is necessary for developing hypotheses regarding adaptive patterns in response to climatic oscillations and elevation gradients [4,5]. Ecological theory suggests that plant performance traits should be associated with the resilience of a species to a particular set of specific weather conditions [6]. For example, leaf morphology varies within species across elevation gradients, and broader leaves are more susceptible to temperature extremes [7,8]. This variation could be indicative of predicting how species will eventually respond to climate change [9]. Current research indicates that climate phenomena are more widespread than previously considered in TMCF [10,11].

Potential impacts of the current temperature oscillation on the distribution and conservation of relict ecosystems are of worldwide concern [12]. Some main consequences of rising temperatures are that several relict–endemic TMCF trees are not morphologically adapted to climate change [12,13]. Consequently, these species could be more vulnerable to reducing leaf–wetting events than others, as they would be more reliant on high moisture conditions.

Between 2019 and 2021, mean maximum temperatures in eastern Mexico by 0.2 °C, whereas mean rainfall rates changed slightly in the study forests [14]. Therefore, certain species of Neotropical *Magnolia* L. trees are better adapted to moisture than others, as they could be more resistant to the hydric deficit because of reduced fog, vapor plumes, mist, or drizzle than those species at lower elevations. Accordingly, we analyzed the leaf vein morphology plasticity in response to elevation gradient that affects the fog rates of four deciduous threatened *Magnolia* species (*M. nuevoleonensis* A. Vázquez & Domínguez-Yescas, *M. alejandrae* García–Morales & Iamonico, *M. rzedowskiana* A. Vázquez, Domínguez–Yescas & Pedraza–Ruiz, and *Magnolia vovidesii* A. Vázquez, Domínguez–Yescas & L. Carvajal) along an elevation gradient (from 1480 to 1894 m asl). These four species exhibit huge leaves (from 20 to 65 cm in length × 15 to 27 cm in width).

Our central hypothesis is that specific leaf vein traits among *Magnolia* species should be significantly less plastic in the lower humid habitat compared to the higher moisture site. Therefore, we addressed the following questions: (1) Are there significant differences between leaf vein (vein density, primary vein size, vein length, and leaf base angle) among four *Magnolia* species restricted to the Mexican tropical montane cloud forests? (2) Is there a leaf vein anatomy adaptation that allows each *Magnolia* species to be resilient to specific climatic factors? (3) Are there specific climatic factors that influence variation in leaf vein traits throughout an elevation gradient?

## 2. Material and Methods

### 2.1. Study Forests

**Figure 1:**
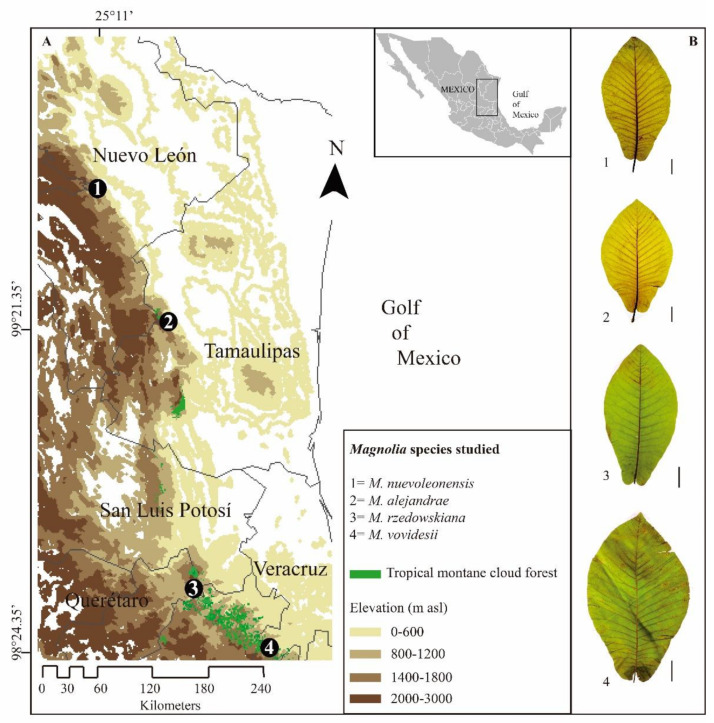
(**A**) Geographic distribution of *Magnolia* species studied in eastern Mexico. (**B**) Leaf morphological variations among species: *Magnolia nuevoleonensis* (1); *M*. *alejandrae* (2); *M*. *rzedowskiana* (3); and *M*. *vovidesii* (4). Black vertical line represents 5 cm in length.

The study was conducted at four Mexican TMCF with different co-dominant *Magnolia* species: (1) La Trinidad locality, Montemorelos municipality, state of Nuevo León (25°11.77′ N, 100°6.84′ W; 1480–1569 m asl); (2) Los San Pedros locality, Güémez municipality, state of Tamaulipas (23°50.91′ N, 99°21.35′ W; 1599–1625 m asl); (3) La Yesca locality, state of Querétaro (21°13′ N, 99°08′ W; 1635–1682 m asl); and (4) El Batda, Huayacotla, Veracruz, Mexico (20°33′ N, 98°24′ W; 1829–1894 m asl), Figure 1. We observed anthropic disturbances at all study sites, from lower to higher degrees (i.e., grazing, logging, coffee and corn plantations, and flower extraction).

1. La Trinidad locality shows a natural distribution (<4 km^2^) of endangered *Magnolia nuevoleonensis* (15–20 m tall), with obovate leaves from 25–35 (–40) × 15–23 (–25) cm [15]. The high-canopy (≤25 m) is composed of *Quercus rysophylla* Weath., *Q*. *pinnativenulosa* C.H. Mull., *Q*. *sartorii* Liebm., *Taxus globosa* Schltdl., *Meliosma alba* (Schltdl.) Walp., *Tilia mexicana* Schltdl., and *Carpinus tropicalis* subsp. *mexicana* Furlow. The study site is affected by deforestation and grazing [16].
2. The Los San Pedros locality is characterized by the presence of critically endangered *M. alejandrae* (8–16 m tall), with obovate leaves from (27–) 30–45 (–55) × 12–20 cm [17]. The study site is dominated by *Quercus rysophylla*, *Q*. *affinis* Scheidw., *Meliosma alba*, *Liquidambar styraciflua* L., *Carpinus tropicalis* (Donn. Sm.) Lundell subsp. *mexicana* Furlow, *Tilia mexicana*, *Ostrya virginiana* subsp. *guatemalensis* (H.J.P. Winkl.) A.E. Murray, *Carya ovata* var. *mexicana* (Engelm.) W.E. Manning, and *Pinus pseudostrobus* Brongn. This is one of the less disturbed *Magnolia* forests in Mexico.
3. La Yesca locality is characterized by the presence of endangered *M*. *rzedowskiana* (8–25 m tall) with ovate-oblong leaves from 25–40 (–50) × 15–25 (–27) cm [18]. The study site is dominated by *Cupressus lusitanica* Mill., *Liquidambar styraciflua*, and *Pinus* spp. The forest is highly disturbed, mainly by grazing, preventing natural regeneration.
4. El Batda locality is characterized by the presence of the endangered *M*. *vovidesii* (8–25 m tall) with ovate-oblong leaves from 25–60 × 16–30 (35) cm [19]. The tree species composition is dominated by *Liquidambar styraciflua*, *Pinus patula* Schltdl. & Cham., *P*. *greggii* Engelm. ex Parl, *Befaria aestuans* L., *Quercus meavei* Valencia-A., Sabás & Soto, *Q*. *delgadoana* S. Valencia, Nixon & L. M. Kelly, and *Q*. *trinitatis* Trel. The mid-canopy is composed of *M*. *schiedeana* Schltdl., *Podocarpus reichei* J. Buchholz & N.E. Gray, *Clethra mexicana* DC., *Dicksonia sellowiana* Hook. var. *arachneosa* Sodiro, *Alsophila firma* (Baker) D.S. Conant, *Cyathea fulva* (M. Martens & Galeotti) Fée, and *Cyathea bicrenata* Liebm. The threatened *Magnolia* species is affected by logging and grazing and is often used in traditional medicine for heart diseases [2].

### 2.2. Leaf Sampling Design

During May–June 2021, 25 fully mature undamaged leaves we collected from the basal branches of 10 mature trees per population on each study site using ropes and arborist-style climbing techniques [20]. The leaves collected were wrapped in a moist newspaper and kept in black plastic bags (60 cm length × 40 cm length) to avoid moisture loss. The leaf samples were transported to the laboratory within two days of collecting.

### 2.3. Leaf-Clearing and Digitalization Methods

**Figure 2:**
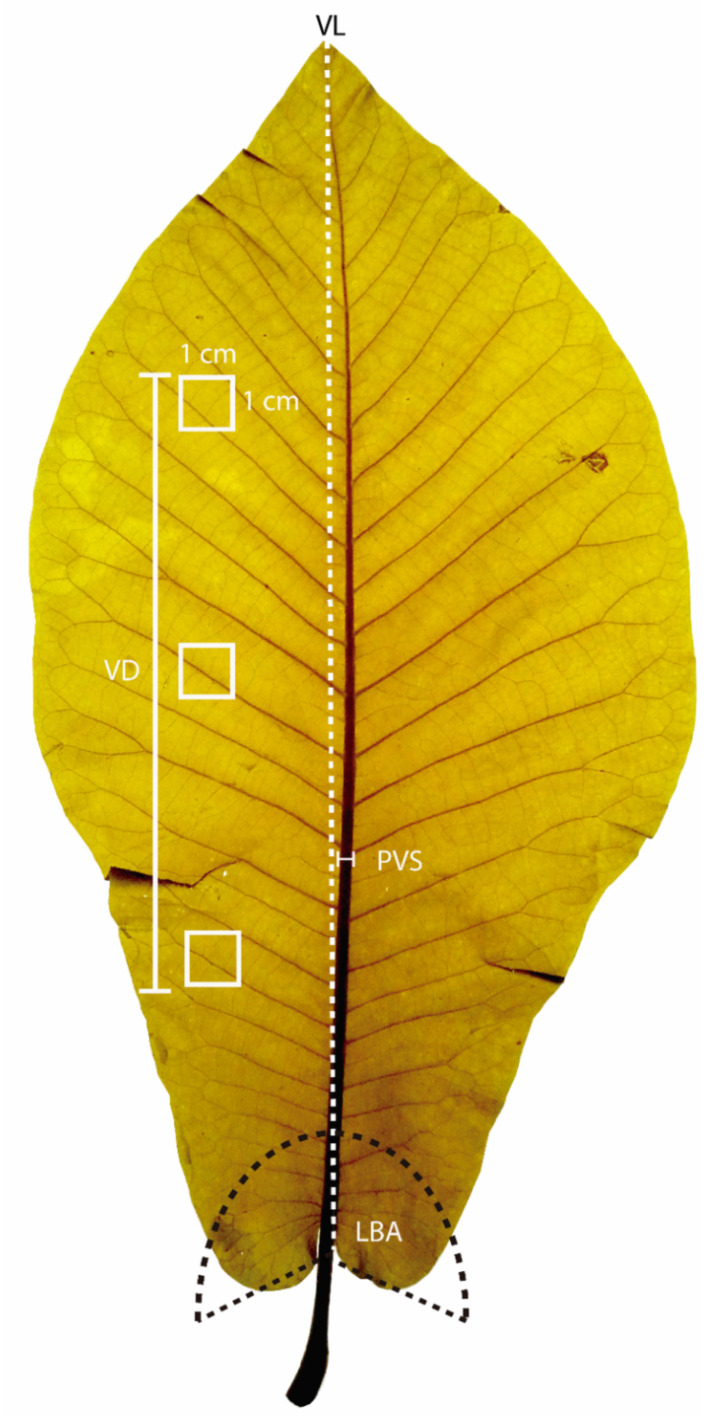
*Magnolia* leaf diagram showing sample vein traits measurements. Vein length (VL), vein density (VD), primary vein size (PVS), and leaf base shape (LBS).

The collected leaves were placed in a container with water and boiled for 45 to 60 min. Subsequently, each leaf was placed on glass (70 × 50 cm) with an opaque background. Then, with the help of a fine brush, the leaf was spread over the entire glass base with a 100–watt lamp to obtain a digital image (600 dpi) with the help of a professional camera (CANON^®^). This method provided high–resolution digital images with uniform illumination, as the resolution was high enough to zoom into the most delicate veins (Figure 2).

### 2.4. Leaf Vein Measurement

**Figure 3:**
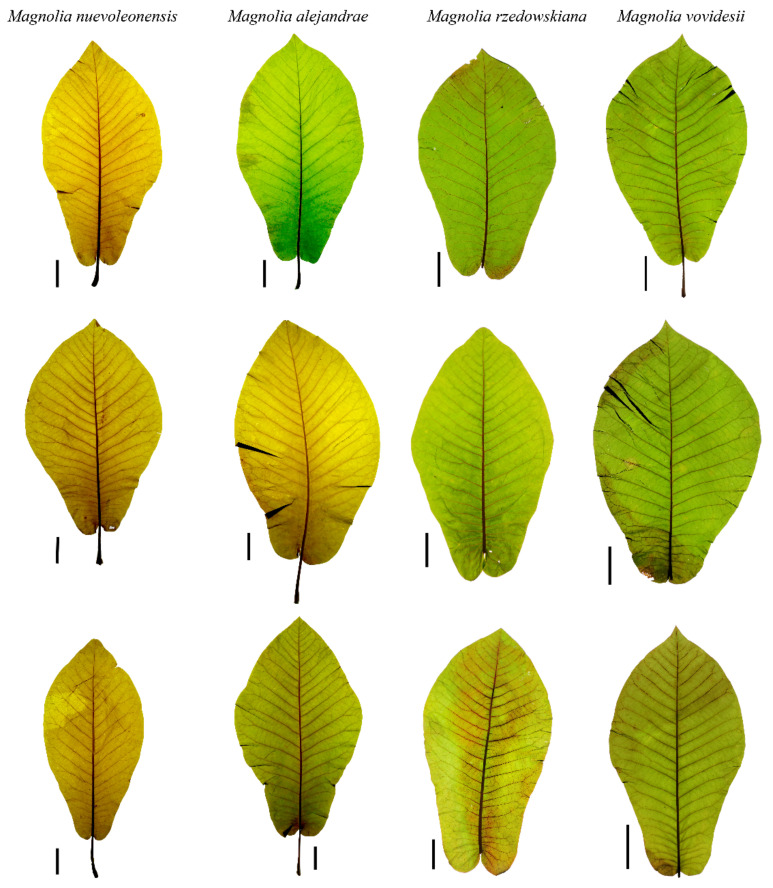
Typical leaf morphological variation among *Magnolia* species. The black horizontal line represents 5 cm.

For each cleared *Magnolia* leaf digitized, four venation traits were measured (accuracy 0.1 mm), related to physiological resilience and hydric adjusting [21]. These included vein density (VD, mm·mm^−2^), primary vein size (PVS, cm), vein length (VL, cm), and leaf base angle (LBA, °). Measurements were performed according to Zhang et al. [22] using software ImageJ v. 1.48 ([23]; available at www.imagej.net, accessed on 20 June 2021). The measurements among *Magnolia* species (Figure 3) were assessed as overlaid bee-swarm with a 95% confidence interval band (R–software v. 4.1.1, *beeswarm*–package) and box-and-whisker plots (*boxplot*–R function) [24].

### 2.5. Climate Data

Maximum temperature (T_max_, °C), minimum temperature (T_min_, °C), average precipitation (P, mm), and potential evaporation (PE, mm) were acquired directly from weather stations near each study site (https://clima.inifap.gob.mx/lnmysr/Estaciones, accessed on 15 September 2021), with climatic records from January to April 2021.

### 2.6. Associating Climatic Factors, Leaf Vein Traits, and Altitude

To evaluate the climatic effect on *Magnolia* leaf vein plasticity, Non-metric Multidimensional Scaling (NMDS; using the Bray–Curtis dissimilarity index), a multivariate analysis with several ecological applications [25], was achieved. First, the stress–plot function and the Stress index were determined to estimate R^2^ values between the leaf vein traits values (vein density, primary vein size, vein length, and leaf base angle) and climate vectors (T_max_, T_min_, P, and PE) of the ordination (R). The vectors for climate data and centroids were then superimposed using the *envfit*–function. Next, the altitude data at the *Magnolia* species was used to create a spatial elevation gradient using the *ordisuf*–function superimposed onto the two NMDS axes [25]. The analysis was performed in R-software using *vegan*–library and *mgcv*–package [26]. Lastly, the estimated error by Generalized Cross-Validation (GCV score; [27]) was assessed.

## 3. Results

### 3.1. Leaf Vein Traits

**Figure 4:**
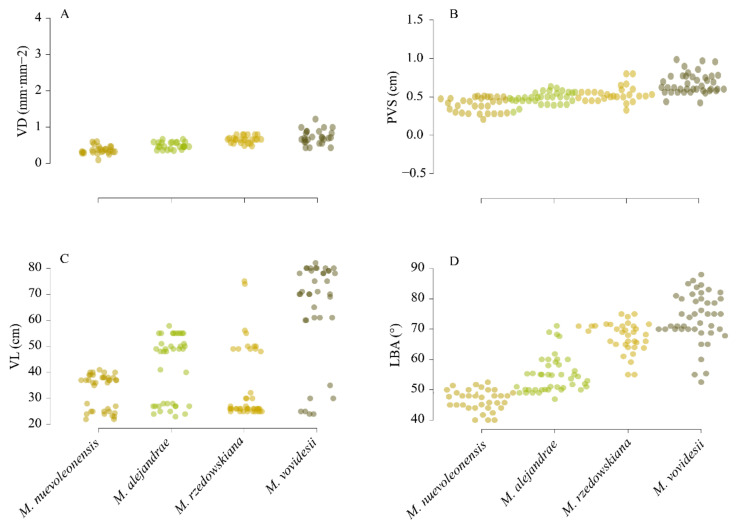
Beeswarm plots displaying leaf vein traits variation among *Magnolia* species. (**A**) vein density; (**B**), primary vein size; (**C**) vein length; and (**D**) leaf base angle.

Even though there were no statistically significant differences, the Spearman test showed the minimum and maximum values among *Magnolia* leaf traits. *Magnolia nuevoleonensis* showed the lowest VD values (from 0.25 to 0.59 mm·mm^−2^) related to *M*. *alejandrae* and *M*. *rzedowskiana*. Notwithstanding, *M*. *vovidesii* had higher VD values (0.43 to 0.99) than the other *Magnolia* species, (Figure 4A). We found that PVS values ranged from 0.28 to 0.81 cm, where *M. nuevoleonensis* showed the lowest PVS values (from 0.28 to 0.5) and *M*. *vovidesii* the highest values (from 0.55 to 0.81), Figure 4B. Vein length (VL) showed significant differences among *Magnolia* species: *M*. *nuevoleonensis* showed the lowest VL values (from 22 to 41), while *M*. *alejandrae* and *M*. *rzedowskiana* observed similar VL values (from 25 to 55) and *M*. *vovidesii* showed higher VL values (from 24 to 80), Figure 4C. The lowest LBA values were detected in *M*. *nuevoleonensis* (from 40 to 50°), and *M*. *alejandrae* and *M*. *rzedowskiana* showed middle LBA values (from 48 to 75). Lastly, *M*. *vovidesii* showed the highest LBA values (from 55 to 88), Figure 4D.

### 3.2. Leaf Vein Traits and Climatic Factors

**Figure 5:**
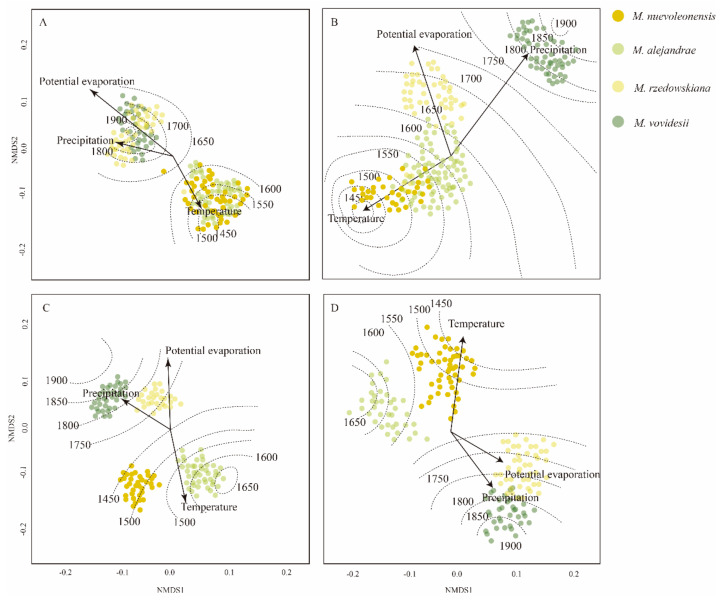
Non-metric Multidimensional Scaling (NMDS) ordination, based on the relationship of climatic factors (T_max_, P and PE) on elevation gradient and leaf vein traits. (**A**) vein density (VD); (**B**) primary vein size (PVS); (**C**) vein length (VL), and (**D**) leaf base angle (LBA). Dotted lines indicate elevation gradients.

Despite the considerable variation in the leaf vein traits among *Magnolia* species, we found clear evidence that interspecific leaf vein traits follow consistent patterns of variation in response to increasing and/or decreasing elevation and local climatic factors in the Mexican TMCFs. *Magnolia nuevoleonensis*, with lower altitude (from 1480 to 1569 m asl), showed lower values for the leaf vein traits measured, while *M*. *alejandrae*, *M*. *rzedowskiana*, and *M*. *vovidesii* showed higher values in leaf vein traits. The Non-metric Multidimensional Scaling showed that temperature had a considerable effect on the VD values of *Magnolia nuevoleonensis* and *M*. *alejandrae* (Figure 5A; Stress index: 0.134; GCV score: 0.028). In turn, precipitation and potential evaporation influenced the VD values of *M*. *rzedowskiana* and *M*. *vovidesii* (Figure 5A). The PVS values of *Magnolia nuevoleonensis* and *M. alejandrae* are influenced by the temperature, while the PVS values of *M*. *rzedowskiana* are affected by the potential evaporation and the ones of *M*. *vovidesii* by high precipitation rates (Figure 5B; Stress index: 0.088; GCV score: 0.028).

The VL is influenced by specific climatic variations in which each of the *Magnolia* species is distributed. For example, the VL values of *M. nuevoleonensis* and *M. alejandrae* are affected by high temperatures, while *M*. *rzedowskiana* and *M*. *vovidesii* are influenced by high precipitations and potential evaporation rates (Figure 5C; Stress index: 0.077; GCV score: 0.047).

The LBA values of *Magnolia nuevoleonensis* are influenced by high temperatures; meanwhile, these LBA values of *M*. *rzedowskiana* and *M*. *vovidesii* are affected by high precipitations and potential evaporation rates (Figure 5D; Stress index: 0.080; GCV score: 0.055). We did not find that LBA of *M*. *alejandrae* was affected by specific climatic factors.

## 4. Discussions and Conclusions

We found that leaf veins significantly vary in the four *Magnolia* species analyzed. Notwithstanding, high leaf vein morphological plasticity occurs among individuals of the same populations. Another kind of variation could occur through gene flow, potential currently unknown [9]. For example, high genetic flow rates among populations nearby (as between *Magnolia nuevoleonensis* and *M*. *alejandrae*, and between *M*. *rzedowskiana* and *M*. *vovidesii*) would contribute to greater phenotypic similarity than by environmental variation influence.

In addition, we found that elevation differences among study sites could be directly or indirectly influencing the *Magnolia* species adaptation and consequently the leaf vein plastic capacity to particular climatic variations [4,28,29]. At higher elevations, as with *Magnolia vovidesii* and *M. rzedowskiana*, high precipitation and potential evaporation promote temperature-tolerant species that invest more carbon on leaf plasticity, as found by Gratani [30] in tropical tree species

The pattern of leaf vein traits showed that leaf size is longest in the southern, wetter, and less arid sites, while leaf size is gradually smaller concerning the northern Mexican TMCFs, in less moist sites. Our observations suggest that *Magnolia vovidesii* and *M*. *rzedowskiana* (with broader leaves) are adapted to high humidity conditions. In comparison, *M*. *nuevoleonensis* and *M*. *alejandrae* (with relatively narrower and smaller leaves) are better adapted to low moisture environments. Broader leaves are widespread in areas with high temperatures and high moisture conditions [31]. Likewise, Williams–Linera [1] mentioned that the width of leaves tends to decrease in open-canopy habitats.

Linking specific climatic factors with leaf vein traits, we can say that *Magnolia* species responded to elevation variations [29] with specific climatic factors such as temperature, precipitation, and potential evaporation rates. Our results suggest that *Magnolia* leaf vein traits decreased leaf size with low moisture conditions and low altitude. Milla and Reich [32] and Rodríguez–Ramírez et al. [29,31] suggested that leaf size decrease occurs in response to local weather and environmental oscillations because these conditions inhibit leaf development, which plays a crucial role in hydric adaptations to precipitation deficit. Therefore, we can suggest that leaf vein traits of *Magnolia nuevoleonensis* and *M*. *alejandrae* are influenced by the increase of elevation and temperature; meanwhile, the leaf vein traits of *M*. *rzedowskiana* and *M*. *vovidesii* are influenced by precipitation and potential evaporation rates.

## Data Availability

Data sharing not applicable.
